# Integrating Navigation-Assisted Ablation in the Locoregional Treatment of Hepatocellular Carcinoma

**DOI:** 10.1001/jamanetworkopen.2024.0694

**Published:** 2024-02-29

**Authors:** Yoshiko Iwai, Chris B. Agala, David A. Gerber

**Affiliations:** 1 University of North Carolina at Chapel Hill School of Medicine, Chapel Hill; 2 Department of Surgery, University of North Carolina at Chapel Hill School of Medicine, Chapel Hill; 3 Lineberger Comprehensive Cancer Center, University of North Carolina at Chapel Hill School of Medicine, Chapel Hill; 4 Now with Department of Surgery, University of Cincinnati College of Medicine, Cincinnati, Ohio

## Abstract

**Importance:**

With the increasing prevalence of hepatocellular carcinoma (HCC), ablative therapy is a critical treatment option to achieve a cancer-free state. The anatomic location of the tumor can be a challenge, and select hepatic locations of a tumor require laparoscopic-assisted strategies to safely reach and treat the lesion.

**Objective:**

To assess the association of real-time ultrasonography-augmented navigation for HCC ablation with patient survival, operative time, and rate of incomplete ablations.

**Design, Setting, and Participants:**

This retrospective case-control study was conducted among a prospectively collected database of more than 750 patients with HCC who were treated with ablation therapy with and without the use of navigation at a single quaternary medical center from June 2011 to January 2021. Data were analyzed from October 2022 through June 2023.

**Exposure:**

Real-time ultrasonography-augmented navigation.

**Main Outcomes and Measures:**

The primary outcome was rate of incomplete ablations in patients undergoing HCC ablation with vs without navigation. Secondary outcomes included overall survival (OS), progression-free survival (PFS), and operative time.

**Results:**

The analytic cohort included 467 patients (mean [SD] age, 62.4 [7.8] years; 355 male [76.0%]; 21 Hispanic [4.5%], 67 non-Hispanic Black [14.5%], and 347 Non-Hispanic White [75.0%] among 463 patients with race and ethnicity data). The most common etiology of liver disease was hepatitis C infection (187 patients with etiology data [40.0%]), and 348 of 458 patients with TMN staging data (76.0%) had TNM stage 1 disease. There were 187 individuals treated with navigation and 280 individuals treated without navigation. Patients who underwent navigation-assisted ablation were more likely to have stage 2 disease based on TNM staging (62 of 183 patients [33.9%] vs 47 of 275 patients [17.1%] with TMN data; *P* < .002) and had a higher mean (SD) number of lesions (1.3 [0.5] vs 1.2 [0.5] lesions; *P* = .002) and a longer mean (SD) operation time (113.2 [29.4] vs 109.6 [32.3] minutes; *P* = .04). Patients who underwent navigation were also more likely to have tumors in segment 8 (59 patients [32.1%] vs 53 of 275 patients with segment data [19.3%] with segment data; *P* = .005) and less likely to have tumors in segment 4 (20 patients [10.9%] vs 54 patients with segment data [19.6%]; *P* = .005). Overall mean (SD) time to recurrence after treatment was 10.0 (12.5) months, with similar rates for patients with navigation vs no navigation. There were no differences in incomplete ablation rate (10 patients [9.2%] vs 10 patients [10.5%]; *P* = .32), OS, or PFS between patients undergoing ablation with and without navigation.

**Conclusions and Relevance:**

In this study, use of navigation was associated with comparable outcomes to undergoing ablation without navigation, although patients with navigation had more locally advanced disease. These findings suggest that use of real-time navigation in laparoscopic-assisted ablation of liver cancer should be considered as a useful tool for treating challenging tumors.

## Key Points

**Question:**

Is navigation associated with efficacy of ablation in the local treatment of hepatocellular carcinoma (HCC)?

**Findings:**

In this case-control study of 467 patients with HCC, there were no statistically significant differences in rate of incomplete ablations or survival outcomes for patients undergoing ablation with vs without navigation, although the navigation group had significantly more patients with locally advanced disease and tumors in more challenging anatomic locations (ie, segment 8).

**Meaning:**

These findings suggest that use of real-time navigation in laparoscopic-assisted ablation of liver cancer should be considered as a useful adjunct for treating challenging tumors.

## Teaser

This case-control study investigates the association of navigation-assisted ablation with rate of incomplete ablations in patients with hepatocellular carcinoma.

## Introduction

Hepatocellular carcinoma (HCC) is the fourth leading cause of cancer-associated mortality in the world.^1^ By 2025, more than 1 million people are anticipated to be annually diagnosed with liver cancer.^1^ While surgical resection continues to be the primary treatment choice for curative intent, most patients are not candidates for resection owing to tumor features, underlying hepatic disease, or comorbid conditions.^2^

The development and implementation of locoregional therapies, including ablative technologies (eg, radiofrequency ablation [RFA] and microwave ablation [MWA]), offers a definitive treatment modality for early-stage, smaller tumors.^3,4,5^ Ablative therapies have evolved over the last 2 decades, showing promise as a robust alternative to surgical resection in select patients.^6,7^ Randomized clinical trials report similar outcomes between surgical resection and RFA, with RFA treatment being less invasive and resulting in shorter hospital stays.^8,9^ Several retrospective studies have reported MWA as a comparable alternative to surgical resection, with minimized complications in a less invasive treatment than an open procedure.^10,11,12,13^ However, an ongoing challenge for ablative therapies involves the technical learning curve for precise localization of the tumor and accurate placement of the device to ensure complete ablation of the lesion while minimizing damage to surrounding structures.^14,15^

Several imaging modalities, including ultrasonography, magnetic resonance imaging (MRI), computed tomography (CT), and fluoroscopy, are actively used to target ablative therapies for HCC.^16^ The concomitant use of CT or MRI guidance is primarily available in the radiology suite and often inaccessible in the operative environment. Thus, many surgical teams use intraoperative ultrasonography to identify the tumor and assist with targeting the ablation probe. However, this approach has technical challenges owing to ultrasonography resolution and anatomic constraints that can limit visualization and is ultimately highly dependent on user experience.^17,18^ With the requisite technical skills required to successfully ablate liver tumors, the introduction of navigation is an exciting development in this domain of minimally invasive surgery. Over the last decade, novel navigation technologies have been developed as an adjunct to intraoperative ultrasonography.^19^

This study analyzes the use of navigation as an adjunct to intraoperative ultrasonography in liver cancer ablation. We hypothesized that the use of navigation would be associated with increased accuracy, a lower rate of incomplete ablations, and thus improved progression-free survival (PFS) compared with the same procedure done without navigation. With the increasing prevalence of HCC disease, ablative therapies for HCC are a vital surgical treatment associated with increased survival at the population level, and navigation is a tool that has the potential for associated increases in accuracy and accessibility of ablation.

## Methods

This case-control study was reviewed by the University of North Carolina Institutional Review Board and approved to operate under a waiver of informed consent because the research involved no more than minimal risk to study participants and could not be practically carried out without the waiver. This study follows the Strengthening the Reporting of Observational Studies in Epidemiology (STROBE) guideline as published.

### Study Design

This is a single-center, retrospective case-control study of a prospectively collected dataset of 757 adult patients (aged ≥18 years) with underlying liver disease who were treated with thermal ablation for HCC at a quaternary academic medical center between June 2001 and January 2021. Study participants were restricted to the time when navigation was introduced at the center (beginning in June 2011). Patient demographics, clinical and pathologic characteristics, and survival were extracted from the electronic health record (EHR). Patient race and ethnicity were identified in the EHR as Asian or Pacific Islander, Hispanic, Native American, non-Hispanic Black, and non-Hispanic White. These were included in the analysis given that there are potential differences in the stage of tumor presentation by race. Data were extracted by a single reviewer (Y.I.) and confirmed by the senior author (D.A.G.). Participants were grouped by whether navigation was used during the ablation. Missing data were assumed to be missing completely at random, thus missingness was assumed to have no effect on results and was ignored in the analysis.

### Surgical Procedure

All procedures were laparoscopic and performed by a single surgeon (D.A.G.) experienced in minimally invasive hepatic surgery. This surgeon has more than 25 years of hepatobiliary surgery experience and more than 20 years of experience performing laparoscopic-assisted liver ablations. The surgeon has incorporated intraoperative navigation for laparoscopic ablation for more than a decade. The comprehensive surgical experience in its entirety includes 1200 tumors treated in more than 750 patients.

All tumors were localized with intraoperative ultrasonography and compared with preoperative MRI or CT scans to confirm location. Lesion number and diameter noted in preoperative imaging were confirmed intraoperatively. MWA procedures were performed with 2 systems. The initial system involved a single or double antenna for the ablation with a power of 45 W for 8 to 10 minutes using a 915-Hz microwave generator (NeuWave System, Ethicon) as per established recommendations. Subsequent MWAs were performed with a 2.45-GHz, 13-G microwave antenna (Emprint SX, Medtronic). The generator was set between 45 and 100 W, and treatment occurred for 5 to 10 minutes based on the projected size of the ablation zone. Track coagulation was used at the end of each treatment. An optimal zone of ablation created a 0.5 to 1.0 cm margin of treated tissue around the tumor.

### Navigation

The Emprint SX Ablation Platform with Thermosphere Technology uses an electromagnetic field generator placed under the patient and spatial sensors to track the location and orientation of the ablation antenna and ultrasonography probe. The antenna sensor is embedded in the antenna shaft itself, and the ultrasonography sensors are clipped onto the head of the ultrasonography probe. Relative instrument locations are rendered as a real-time augmented reality image providing additional spatial information. Reference ablation volumes are derived from planned wattage and duration of ablation. These can be projected on the ultrasonography image to visualize and plan for the size, position, and extent of the ablation zone before it is initiated. Volumes are based on preclinical in vivo animal models. Targeting requires the user to aim the antenna at the location identified in the ultrasonography image. A small, green circle illuminates the antenna’s projected path to the target on the ultrasonography plane. Once the image of the antenna is lined up, the user advances the antenna to the target. This 3-dimensional, real-time spatial data allows the surgeon to predetermine and approach a target from various out-of-plane paths and subsequently advance with line-of-sight trajectory (Video).

**Video.** Recording of Navigation-Assisted Laparoscopic Ablation of a Liver Lesion Video recording of navigation use for laparoscopic ablation of a liver lesion. The location and orientation of the ablation antenna and ultrasonography probe are visualized to provide the surgeon with 3-dimensional real-time spatial data to predetermine and approach a target.

### Statistical Analysis

The primary outcome of interest was the rate of incomplete ablations. Secondary outcomes included PFS, overall survival (OS), and operative time. Patient, disease, and treatment characteristics are summarized with median (IQR) or mean (SD) for continuous variables and number (percentage) for categorical variables by navigation use. Differences between groups (navigation vs no navigation) were tested using the Wilcoxon rank sum test for continuous variables and the χ^2^ or Fisher exact test for categorical variables. Normality tests (Shapiro-Wilk test and visual inspection of histograms) were conducted to determine if nonparametric methods were appropriate for between-group comparisons of continuous variables. The Shapiro-Wilk test checks for normality assumption using W statistics, which are positive and less than or equal to 1. Small values of W less than 0.05 indicate nonnormal distribution.

Crude 5-year OS and PFS were estimated using the Kaplan-Meier method, and differences between groups were tested using the log-rank test. The Kaplan-Meier method is a nonparametric maximum likelihood estimator of the survival function for censored data. This analysis produces survival curves in the postoperative period, which shows the estimated survival probability for each time by group on the y-axis and follow-up time intervals on the x-axis. Data for all cases were used in this analysis until the end of follow-up or occurrence of the event of interest (recurrence or death). Cox proportional hazards models were used to estimate hazard ratios (HRs) for associations between independent factors and OS and PFS. The Cox proportional hazard model is a semiparametric maximum likelihood regression method for assessing associations between 1 or more factors hypothesized to be associated with the outcome and survival time to events of interest (recurrence or death). All independent factors in all models were selected a priori based on clinical knowledge and experience.

A nominal significance level of .05 was used for all statistical tests. Statistical analyses were conducted using SAS statistical software version 9.4 (SAS Institute). Data were analyzed from October 2022 through June 2023.

## Results

### Patient Characteristics

From the initial set of 757 total records, 666 patients met criteria for potential inclusion. The final cohort was restricted to the time when navigation became available as a technology at the study institution (ie, 2011), for a total of 467 patients (mean [SD] age, 62.4 [7.8] years; 355 male [76.0%]; 21 Hispanic [4.5%], 67 non-Hispanic Black [14.5%], and 347 Non-Hispanic White [75.0%] among 463 patients with race and ethnicity data), including 187 patients who received navigation and 280 patients who received no navigation (eFigure 1 in Supplement 1). Patient demographics are summarized in Table 1. The etiology of liver disease was most commonly hepatitis C infection (187 patients [40.9%]), followed by metabolic-associated fatty liver disease (98 patients [21.4%]) and a combination of hepatitis C infection and alcohol cirrhosis (97 patients [21.2%]) among 457 patients with etiology data. The breakdown of TNM staging was 348 patients (76.0%) with stage 1, 109 patients (23.8%) with stage 2, and 1 patient (0.2%) with stage 3A disease among 458 patients with TMN data. Ablation was performed using several systems: Medtronic Emprint MWA (264 patients [57.4%]), Covidien MWA (66 patients [14.4%]), and Ethicon NeuWave MWA (121 patients [26.3%]) among 460 patients with ablation system data. Model for End-Stage Liver Disease (MELD) scores were most commonly between 5 and 11 (312 of 445 patients [70.1%] with data), while Child-Pugh (CP) scores were most commonly between 5 and 6 (264 of 450 patients [58.7%] with data), and α-fetoprotein tumor marker (AFP) levels were most commonly 20 ng/mL or less (284 of 405 patients [70.1%] with data) (to convert AFP to micrograms per liter, multiply by 1.0).

**Table 1.** Patient Characteristics

Characteristic	Patients, No. (%)			*P* value
	All patients (N = 467)	No navigation (n = 280)	Navigation (n = 187)	
Age, mean (SD), y	62.4 (7.8)	62.2 (8.3)	62.7 (7.0)	.65
Sex				
Female	112 (23.98)	67 (23.05)	45 (24.06)	>.99
Male	355 (76.02)	213 (76.95)	142 (75.94)	
Race				
Total with data, No.	463	277	186	NA
Asian or Pacific Islander	17 (3.67)	9 (3.25)	8 (4.30)	.12
Hispanic	21 (4.54)	15 (5.42)	6 (3.23)	
Native American	8 (1.73)	2 (0.72)	6 (3.23)	
Non-Hispanic Black	67 (14.47)	36 (13.00)	31 (16.67)	
Non-Hispanic White	347 (74.95)	212 (76.53)	135 (72.58)	
Unknown	3 (0.65)	3 (1.08)	0	
Ablation system				
Total with data, No.	460	273	187	NA
AngioDynamics Microsulis	9 (1.96)	0	9 (4.81)	<.001
Covidien	66 (14.35)	33 (12.09)	33 (17.65)	
Medtronic Emprint	264 (57.39)	187 (68.5)	77 (41.18)	
Ethicon NeuWave	121 (26.30)	53 (19.41)	68 (36.36)	
Etiology				
Total with data, No.	457	272	185	NA
Autoimmune hepatitis	4 (0.88)	3 (1.10)	1 (0.54)	.84
Cryptogenic	8 (1.75)	5 (1.84)	3 (1.62)	
Alcohol use	66 (14.44)	40 (14.71)	26 (14.05)	
Hemochromatosis	1 (0.22)	1 (0.37)	0	
Hepatitis B	13 (2.84)	7 (2.57)	6 (3.24)	
Hepatitis C	187 (40.92)	103 (37.87)	84 (45.41)	
Hepatitis C and EtOH	77 (16.85)	50 (18.38)	27 (14.59)	
MAFLD	98 (21.44)	61 (22.43)	37 (20)	
Primary biliary cholangitis	3 (0.66)	2 (0.74)	1 (0.54)	
CT MRI stage				
0	8 (1.71)	7 (2.50)	1 (0.53)	.001
1	352 (75.37)	224 (80.00)	128 (68.45)	
2	85 (18.20)	40 (14.29)	45 (24.06)	
3	15 (3.21)	4 (1.43)	11 (5.88)	
4	7 (1.50)	5 (1.79)	2 (1.07)	
TNM stage				
Total with data, No.	458	275	183	NA
1	348 (75.98)	227 (82.55)	121 (66.12)	<.001
2	109 (23.8)	47 (17.09)	62 (33.88)	
3A	1 (0.22)	1 (0.36)	0	
Recurrence location				
Total with data, No.	180	100	80	NA
<0.5 cm from ablation zone	84 (45.41)	47 (45.19)	37 (45.68)	.71
≥0.5 cm from ablation zone	96 (51.89)	53 (50.96)	43 (53.09)	
Extrahepatic	4 (2.16)	3 (2.88)	1 (1.23)	
MELD score				
Total with data, No.	445	265	180	NA
5-11	312 (70.11)	187 (70.57)	125 (69.44)	.59
12-20	122 (27.42)	70 (26.42)	52 (28.89)	
21-30	11 (2.47)	8 (3.02)	3 (1.67)	
CP score				
Total with data, No.	450	269	181	NA
5-6	264 (58.67)	153 (56.88)	111 (61.33)	.64
7-9	147 (32.67)	92 (34.2)	55 (30.39)	
10-15	39 (8.67)	24 (8.92)	15 (8.29)	
AFP level, ng/mL				
Total with data, No.	405	250	155	NA
AFP≤20	284 (70.12)	187 (74.8)	97 (62.58)	.04
AFP = 20-99	62 (15.31)	29 (11.6)	33 (21.29)	
AFP = 00-399	36 (8.89)	20 (8.00)	16 (10.32)	
AFP≥400	23 (5.68)	14 (5.6)	9 (5.81)	

Abbreviations: AFP, α-fetoprotein tumor marker; CP, Child-Pugh; CT, computed tomography; MAFLD, metabolic associated fatty liver disease; MELD, Model for End-Stage Liver Disease; MRI, magnetic resonance imaging.

SI conversion factor: To convert AFP to micrograms per liter, multiply by 1.0.

Patients who underwent navigation-assisted ablation were more likely than patients with no navigation to have advanced disease based on TNM staging (stage 2 disease: 62 of 183 patients [33.9%] vs 47 of 275 patients [17.1%] with TMN data; *P* < .002) and MRI staging (stage 2 disease: 45 patients [24.1%] vs 40 patients [14.3%]; stage 3 disease: 11 patients [5.9%] vs 4 patients [1.4%]; *P* = .001). Patients in the navigation group were also more likely to undergo ablation when using the NeuWave MWA (68 of 187 patients [36.4%] vs 53 of 273 patients with data [19.4%]) and Covidien MWA systems (33 patients [17.7%] vs 33 patients [12.1%]) but less likely to undergo ablation with the Metronic Emprint MWA system (77 patients [41.2%] vs 187 patients [68.5%]; *P* < .001) compared with patients with no navigation. There were no differences between navigation and no navigation groups for age, sex, race and ethnicity, disease etiology, recurrence location, MELD score, CP score, or AFP level (Table 1).

### Tumor Characteristics

Tumor characteristics for patients undergoing ablation with and without navigation are summarized in Table 2. The overall cohort had a mean (SD) total bilirubin level of 1.6 (1.3) mg/dL, albumin level of 3.5 (0.6) g/dL, international normalized ratio of 1.2 (0.2) seconds, platelet count of 117.6 (68.1) × 10^9^/L, and creatinine level of 0.9 (0.4) mg/dL (Table 2). (To convert bilirubin to micromoles per liter, multiply by 17.104; albumin to grams per liter, multiply by 10.0; platelet count to ×109/L, multiply by 1.0; and creatinine to micromoles per liter, multiply by 88.4.) The mean (SD) MELD score was 10.4 (3.9). The mean (SD) number of liver lesions was 1.2 (0.5) lesions, with a mean size of 2.3(0.8) cm. The mean (SD) operating time was 110.9 (31.3) minutes, with an ablation time of 15.1 (6.7) minutes. The mean (SD) number of recurrent tumors was 1.5 (1.0) tumors, with a mean (SD) recurrent tumor size of 2.5 (1.6) cm. Tumors were most frequently located in segment 8 (112 patients [24.4%]), followed by segment 4 (74 patients [16.1%]), segment 7 (70 patients [15.3%]), segment 6 (69 patients [15.0%]), and segment 2 (54 patients [11.8%]) among 459 patients with segment data (eTable 1 in Supplement 1). Overall, incomplete ablations were recorded in 20 of 459 patients with ablation data (9.8%).

**Table 2.** Tumor Characteristics

Characteristic	All patients (N = 467)		No navigation (n = 280)		Navigation (n = 187)		*P* value
	Patients, No.	Mean (SD)	Patients, No.	Mean (SD)	Patients, No.	Mean (SD)	
Total bilirubin, mg/dL	461	1.6 (1.3)	276	1.6 (1.3)	185	1.6 (1.2)	.78
Albumin, g/dL	460	3.5 (0.6)	275	3.6 (0.6)	185	3.5 (0.6)	.87
INR, sec	455	1.2 (0.2)	271	1.2 (0.3)	184	1.2 (0.2)	.82
Platelet count, ×10^9^/L	462	117.6 (68.1)	276	118.0 (68.3)	186	117.0 (68.0)	.99
Creatinine, mg/dL	462	0.9 (0.4)	277	0.9 (0.5)	185	0.9 (0.3)	.75
No. of lesions	434	1.2 (0.5)	259	1.2 (0.5)	175	1.3 (0.5)	.003
Lesion diameter, cm	61	2.3 (0.8)	58	2.3 (0.8)	3	2.0 (1.0)	.54
Recurrence	135	1.5 (1.0)	71	1.3 (0.7)	64	1.8 (1.2)	.11
Recurrent tumor size, cm	83	2.5 (1.6)	30	2.7 (1.2)	53	2.4 (1.8)	.02
Operation time, min	412	110.9 (31.3)	254	109.6 (32.3)	158	113.2 (29.4)	.04
Ablation time, min	453	15.1 (6.7)	270	15.3 (6.6)	183	14.9 (6.8)	.44

Abbreviation: INR, international normalized ratio.

SI conversion factors: To convert albumin to grams per liter, multiply by 10.0; bilirubin to micromoles per liter, multiply by 17.104; creatinine to micromoles per liter, multiply by 88.4; platelet count to ×109/L, multiply by 1.0.

Patients who underwent navigation-assisted ablation had a higher mean (SD) number of lesions (1.3 [0.5] vs 1.2 [0.5] lesions; *P* = .002) and a longer mean (SD) operative time (113.2 [29.4] vs 109.6 [32.3] minutes; *P* = .04) compared with patients with no navigation. On subanalysis, patients who had more than 1 tumor had longer mean (SD) operative times than those with 1 tumor in navigation (121.9 [21.8] vs 111.6 [28.2] minutes; *P* = .02) and no navigation (119.1 [28.8] vs 107.5 [30.8] minutes; *P* = .04) groups (eTable 2 in Supplement 1).

Patients who underwent navigation were more likely than patients with no navigation to have tumors in segment 8 (59 of 184 patients [32.1%] vs 53 of 275 patients [19.3%] with segment data; *P* = .005) and less likely to have tumors in segment 4 (20 patients [10.9%] vs 54 patients [19.6%]; *P* = .005). A subanalysis of tumors located in anatomically challenging segments (ie, segments 6-8) is reported in eTable 3 in Supplement 1. There were no statistically significant differences in recurrence rates or incomplete ablation rates among patients undergoing ablations with vs without navigation, irrespective of whether the lesion was in a challenging location. We defined challenging location based on liver segments, with segment 6 being a moderately challenging location due to dysmorphic features of the cirrhotic liver and its location next to the abdominal wall and segments 7 and 8 being the most challenging location due to their cephalad and posterior anatomy. Among patients with lesions in segments 1 to 5, there was a statistically significant difference in rates of death among patients undergoing ablation with vs without navigation; however, this difference was not appreciable when challenging segments were expanded to include segment 6.

There were no differences in incomplete ablation rates (10 patients [9.2%] vs 10 patients [10.5%]; *P* = .32) or tumor size among patients undergoing ablation with vs without navigation. The overall rate of incomplete ablations trended downward over the course of the study (eFigure 2 in Supplement 1).

### Clinical Outcomes

Clinical outcomes among patients who underwent ablation with and without navigation are summarized in eTable 4 in Supplement 1. A total of 79 patients (16.9%) underwent orthotopic liver transplant after ablation. The study cohort readmission rate was 18 of 205 patients (8.8%) with readmission data. The overall recurrence rate was 195 patients (41.8%) with recurrence data, and 169 patients (36.2%) were deceased at the time of data collection. The rate of recurrence trended downward over the study period (eFigure 2 in Supplement 1).

**Figure 1.**
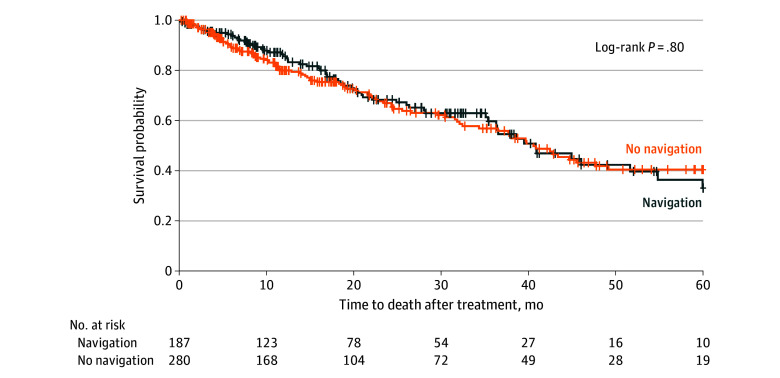
Kaplan-Meier Plot for 5-y Overall Survival Plus signs indicate censored patients.

**Figure 2.**
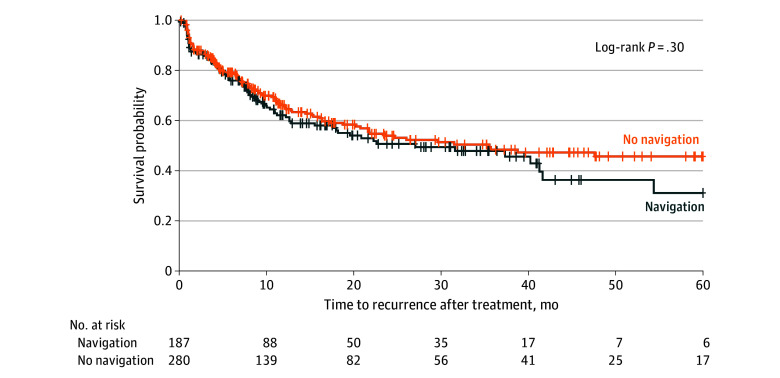
Kaplan-Meier Plot for 5-y Recurrence-Free Survival Plus signs indicate censored patients.

The most frequent cause of death was unknown (80 patients [48.2%]), followed by liver failure (60 patients [36.1%]), among 166 patients with cause of death data. Overall mean (SD) time to recurrence after treatment was 10.0 (12.5) months, with similar rates for patients undergoing ablation with vs without navigation (10.5 [13.6] vs 9.8 [12.5] months; *P* = .96). Mean (SD) time to death after treatment was 24.1 (25.5) months, with no significant difference between navigation (26.7 [24.0] months) and no navigation (20.3 [19.2] months) groups (*P* = .09). Mean (SD) time to orthotopic liver transplant after treatment was 8.7 (6.7) months overall and similar among patients who received navigation (10.3 [6.4] months) vs no navigation (9.7 [7.4] months) (*P* = .31). On Kaplan-Meier estimation, there were no statistically significant differences in OS (*P* = .80) (Figure 1) or PFS (*P* = .30) (Figure 2).

HRs for recurrence are summarized in Table 3. Female sex was associated with a lower likelihood of recurrence, controlling for liver transplantation and death (HR, 0.64 [95% CI, 0.42-0.99]; *P* = .04). Having a higher CP score was associated with a lower likelihood of recurrence comparing CP scores of 10 to 15 vs 5 to 6 (HR, 0.39 [95% CI, 0.19-0.72]; *P* = .01) and 7 to 9 vs 5 to 6 (HR, 0.58 [95% CI, 0.39-0.85]; *P* = .005). There were no statistically significant differences in likelihood of recurrence between patients undergoing ablation with navigation vs no navigation or by age, ablation system, MRI or TNM stage, tumor segment, number of lesions, or AFP level. For the 5-year HR for recurrence, controlling for liver transplantation and death, only CP score was associated with lower likelihood of recurrence, for CP scores 10 to 15 vs 5 to 6 (HR, 0.35 [95% CI, 0.16-0.77]; *P* = .01) and 7 to 9 vs 5 to 6 (HR 0.59 [95% CI, 0.40-0.87]; *P* = .007).

**Table 3.** Risk of Recurrence, Controlling for Liver Transplant and Death as Competing Risks (N = 467)

Characteristic	Comparison	HR (95% CI)	*P* value
Navigation	Navigation vs no navigation	0.87 (0.61-1.24)	.45
Age	Per 1-y increase	1 (0.98-1.03)	.92
Sex	Female vs male	0.64 (0.42-0.99)	.04
Ablation system	AngioDynamics Microsulis vs Medtronic Emprint	1.07 (0.43-2.67)	.89
	Boston Scientific vs Medtronic Emprint	NA^a^	NA^a^
	Covidien vs Medtronic Emprint	1.27 (0.74-2.17)	.39
	Ethicon NeuWave vs Medtronic Emprint	1.35 (0.90-2.02)	.14
	RITA vs Medtronic Emprint	NA^a^	NA^a^
CT MRI stage	Per 1-SD increase	1.12 (0.82-1.53)	.49
TNM stage	2 vs 1	0.90 (0.54-1.52)	.70
	3A vs 1	1.70 (0.77-3.76)	.19
Segment	1 vs 8	1.74 (0.14-21.57)	.67
	2 vs 8	0.80 (0.44-1.44)	.45
	3 vs 8	0.56 (0.24-1.29)	.17
	4 vs 8	0.82 (0.46-1.46)	.50
	5 vs 8	0.87 (0.51-1.48)	.60
	6 vs 8	0.69 (0.40-1.18)	.18
	7 vs 8	0.75 (0.44-1.29)	.30
No. of lesions	Per 1-SD increase	1.08 (0.66-1.76)	.77
AFP level	20-99 vs ≤20	1.30 (0.82-2.06)	.27
	100-399 vs ≤20	1.38 (0.78-2.42)	.27
	≥400 vs ≤20	0.98 (0.48-1.97)	.94
CP score	10-15 vs 5-6	0.39 (0.19-0.72)	.01
	7-9 vs 5-6	0.58 (0.39-0.85)	.005

Abbreviations: AFP, α-fetoprotein tumor marker; CP, Child-Pugh; CT, computed tomography; MRI, magnetic resonance imaging; NA, not applicable; RITA, radiofrequency interstitial tissue ablation.

^a^ Comparison involved patients who were in the initial total pool but not in the analysis set because navigation was introduced after these ablation systems were no longer in use.

## Discussion

This case-control study assessed the association of real-time ultrasonography-augmented navigation for HCC ablation with patient survival, operative time, and rate of incomplete ablations. Intraoperative navigation was associated with comparable patient survival outcomes in patients who did not undergo navigation despite being performed in higher-stage HCC disease and anatomically challenging liver segments (eg, segments 6-8).^20,21^ Intraoperative time was longer for patients undergoing ablation with navigation, but rates of incomplete ablations were similar among patients who did vs did not receive navigation assistance. These findings complement the existing literature on the use of ablation in the treatment of hepatic tumors.^22^

To our knowledge, this study is the first of its kind to compare the integration of navigation with laparoscopic-assisted ablation of HCC. The potential implications for the role of intraoperative navigation in treating anatomically challenging liver tumors is far reaching. The current literature on navigation integration with laparoscopic ablation is limited. In a 2022 study by Ohama et al,^23^ the use of ultrasonography-guided RFA for initial recurrence of early-stage HCC was reported to be of equal therapeutic efficacy compared with resection. Our study’s finding of a comparable rate of recurrence despite differences in tumor stage and location suggests that navigation may fill a unique and important gap in treating HCC among patients who are not candidates for surgical resection.

One of the challenges with laparoscopic ablation of liver tumors is the difficulty in locating lesions in difficult-to-reach anatomical locations of the liver. In this study, tumors located in the dome (eg, segments 7 and 8) were more readily accessed via navigation. The advantage of navigation assistance may be more pronounced when surgeons are still relatively new to laparoscopic ablation. The steep learning curve for laparoscopic liver tumor ablation is well documented and remains a barrier for broad adoption of the technique, despite its potential benefits.^15^ The learning curve for laparoscopic ablation ranges from approximately 50 to 93 cases to reach significant reductions in an incomplete ablation rate for malignant liver tumors^15,24,25^; however, literature on how navigation affects this learning curve is lacking. While measuring the learning curve was outside the scope of this study, the overall downtrend in rates of incomplete ablations and recurrences seen over our study period suggest that navigation may allow for a shorter learning curve. In addition to dedicated training, the incorporation of navigation into laparoscopic ablation training may help flatten the learning curve, leading to broader adoption.

We initially hypothesized that incorporating navigation would be associated with improved targeting accuracy, with a lower rate of incomplete ablations compared with the same procedure done without navigation. This hypothesis was not supported by results, and we suspect that the lack of change in the rate of incomplete ablations was associated with several factors. First, the surgeon performing the procedure had more than a decade of experience with laparoscopic ablations when navigation technology was introduced. At this point in the surgeon’s experience, there may be less benefit to gain in targeting accuracy, and it was challenging to control for this human-factor in the study. Another factor that may be contributing to the lack of an association with incomplete ablations is selection bias. Tumors that were preoperatively identified as being in an accessible location did not routinely have navigation technology incorporated in the case at the study institution, thus introducing bias where navigation was used for lesions located in more challenging anatomic locations (eg, posterior and superior locations). We did, however, see that incomplete ablation rates improved over time, potentially associated with progressive improvement in technical experience. We suspect that if the study group had been larger and conducted over a longer period, there may have been statistically significant differences in the rates of incomplete ablations.

An unexpected finding in the analysis of PFS was that higher CP scores were protective for recurrence compared with lower CP scores. Our finding that patients with higher CP scores had a lower risk of recurrence may be attributed to selection bias, in which patients with more severe illness may not have been offered locoregional therapy for HCC. It is also possible that our findings are a result of incomplete data reporting, in which patients with recurrent disease could present at a local hospital due to the progression of their liver disease and die without the tertiary center obtaining information regarding their cause of death.

Overall, findings of this study suggest a promising and important role of navigation in the treatment of liver cancer. The use of navigation as an operative adjunct is increasing in medicine, and this study suggests potential for the translation of these innovations to locoregional tumor treatment in the future.

### Limitations

This study has several limitations. It was completed at a single center, and the generalizability of our findings is difficult to ascertain. Because the procedure was performed by a surgeon who had more than 20 years of operative experience, there may be temporal trends (eg, technical proficiency) that were not captured by our methods. Selection bias may have affected our study findings as previously mentioned regarding CP scores, recurrence rates, and higher rates of segment 8 lesions among patients undergoing navigation. Data were collected manually from EHRs, and while members of the study team confirmed any discrepancies, the data collection process was subject to human error. Additionally, this is a retrospective study, and limitations include those that are inherent to this study design.

## Conclusions

In this case-control study of intraoperative ultrasonography navigation for liver cancer ablation, the use of navigation was associated with comparable outcomes among patients undergoing ablation with and without navigation, despite treating patients with more locally advanced disease and difficult-to-reach tumors. Additionally, our findings suggest that incorporating navigation may be associated with shortened learning curves and thus broader adoption of laparoscopic ablation for HCC. This study’s results suggest that the use of real-time navigation in laparoscopic-assisted ablation of liver cancer should be considered as a tool for treating challenging tumors.

## Notes

Supplement 1. **eFigure 1.** Flowchart of Patient Inclusion in the Study **eTable 1.** Tumor Locations and Incomplete Ablation Rates **eTable 2.** Operative Times **eTable 3.** Rates of Recurrence, Death, and Incomplete Ablations **eFigure 2.** Rates of Incomplete Ablations and Recurrences by Year **eTable 4.** Clinical Outcomes

Supplement 2.
Data Sharing Statement
