# Supplementary material for: Integrating Navigation-Assisted Ablation in the Locoregional Treatment of Hepatocellular Carcinoma
Source: JAMA Netw Open. 2024 Feb 29;7(2):e240694. doi: 10.1001/jamanetworkopen.2024.0694 (PMC10905302; doi:10.1001/jamanetworkopen.2024.0694)
Supplement: Supplement 1. — eFigure 1. Flowchart of Patient Inclusion in the Study eTable 1. Tumor Locations and Incomplete Ablation Rates eTable 2. Operative Times eTable 3. Rates of Recurrence, Death, and Incomplete Ablations eFigure 2. Rates of Incomplete Ablations and Recurrences by Year eTable 4. Clinical Outcomes [file jamanetwopen-e240694-s001.pdf]

Supplemental Online Content

Iwai Y, Agala CB, Gerber DA. Integrating navigation-assisted ablation in the locoregional treatment of hepatocellular carcinoma. *JAMA Netw Open*. 2024;7(2):e240694. doi:10.1001/jamanetworkopen.2024.0694

eFigure 1. Flowchart of Patient Inclusion in the Study
eTable 1. Tumor Locations and Incomplete Ablation Rates
eTable 2. Operative Times
eTable 3. Rates of Recurrence, Death, and Incomplete Ablations
eFigure 2. Rates of Incomplete Ablations and Recurrences by Year
eTable 4. Clinical Outcomes

This supplemental material has been provided by the authors to give readers additional information about their work.

© 2024 Iwai Y et al. *JAMA Network Open.*

Supplement 1. eFigure 1. Flow diagram of patient inclusion in the study

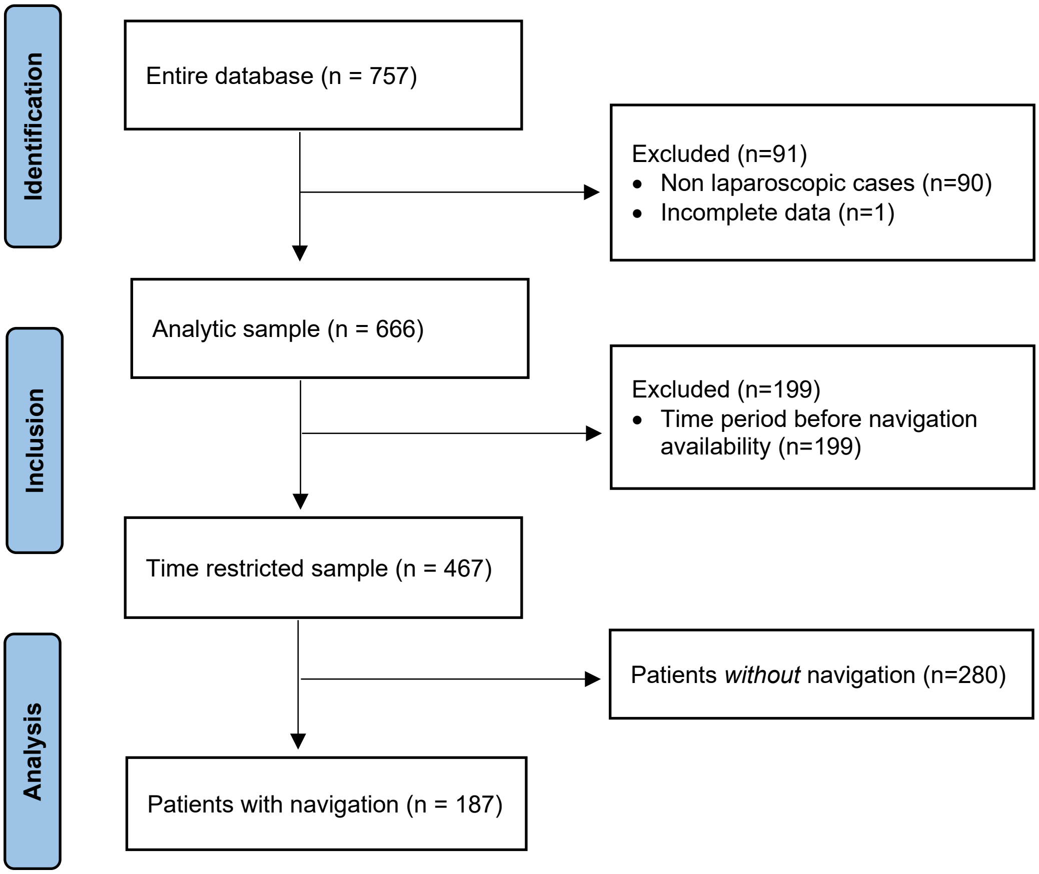

© 2024 Iwai Y et al. *JAMA Network Open.*

Supplement 2. eTable 1. Tumor locations and incomplete ablation rates for patients undergoing ablative therapy for hepatocellular carcinoma with navigation vs without navigation, 2011-2022

	**All patients (n=467)**	**No navigation (n=280)**	**Navigation (n=187)**	**p-value**
**Characteristic**	**No. (%)**	**No. (%)**	**No. (%)**	
Segment				
1	3 (0.65)	2 (0.73)	1 (0.54)	0.005
2	54 (11.76)	34 (12.36)	20 (10.87)	
3	22 (4.79)	19 (6.91)	3 (1.63)	
4	74 (16.12)	54 (19.64)	20 (10.87)	
5	55 (11.98)	33 (12.0)	22 (11.96)	
6	69 (15.03)	42 (15.27)	27 (14.67)	
7	70 (15.25)	38 (13.82)	32 (17.39)	
8	112 (24.40)	53 (19.27)	59 (32.07)	
Incomplete ablation				
No	184 (90.2)	85 (89.47)	99 (90.83)	0.75
Yes	20 (9.8)	10 (10.53)	10 (9.17)	

Supplement 3. eTable 2. Operative times of patients undergoing ablative therapy for hepatocellular carcinoma with navigation vs without navigation grouped by 1 vs >1 tumors, 2011-2022

	**N**	**Mean (minutes)**	**Std Dev (minutes)**	**p-value**
**Operative times for patients without navigation group**				
1 tumor	197	107.5	30.8	0.0392
At least 2 tumors	36	119.1	28.8	
**Operative times for patients with navigation group**				
1 tumor	107	111.6	28.2	0.022
At least 2 tumors	43	121.9	21.8	
**Operative times for patients irrespective of navigation group**				
1 tumor	304	109	29.9	0.0006
At least 2 tumors	79	120.6	25.1	

*p-values based on the Wilcoxon rank sum test

© 2024 Iwai Y et al. *JAMA Network Open.*

Supplement 4. eTable 3. Rates of recurrence, death, and incomplete ablations among patients undergoing ablative therapy for hepatocellular carcinoma with navigation vs without navigation grouped by lesions in challenging liver segments, 2011-2022

	**Segments 1-5**				**Segments 6-8**			
**Treatment method**	**No. (%)**	**No. (%)**	**No. (%)**	**p-value**	**No. (%)**	**No. (%)**	**No. (%)**	**p-value**
	**Overall**	**No recurrence**	**Recurrence**		**Overall**	**No recurrence**	**Recurrence**	
No navigation	142 (68.27)	86 (69.35)	56 (66.67)	0.76	133 (52.99)	79 (56.03)	54 (49.09)	0.31
Navigation	66 (31.73)	38 (30.65)	28 (33.33)		118 (47.01)	62 (43.97)	56 (50.91)	
	**Overall**	**Survived**	**Died**		**Overall**	**Survived**	**Died**	
No navigation	142 (68.27)	92 (73.6)	50 (60.24)	**0.049**	133 (52.99)	87 (52.1)	46 (54.76)	0.79
Navigation	66 (31.73)	33 (26.4)	33 (39.76)		118 (47.01)	80 (47.9)	38 (45.24)	
	**Overall**	**No Incomplete Ablation**	**Incomplete Ablation**		**Overall**	**No Incomplete Ablation**	**Incomplete Ablation**	
No navigation	142 (68.27)	137 (68.84)	5 (55.56)	0.47	133 (52.99)	128 (53.33)	5 (45.45)	0.76
Navigation	66 (31.73)	62 (31.16)	4 (44.44)		118 (47.01)	112 (46.67)	6 (54.55)	
	**Segments 1-6**				**Segments 7-8**			
**Treatment method**	**No. (%)**	**No. (%)**	**No. (%)**	**p-value**	**No. (%)**	**No. (%)**	**No. (%)**	**p-value**
	**Overall**	**No recurrence**	**Recurrence**		**Overall**	**No recurrence**	**Recurrence**	
No navigation	184 (66.43)	113 (67.26)	71 (65.14)	0.80	91 (50)	52 (53.61)	39 (45.88)	0.37
Navigation	93 (33.57)	55 (32.74)	38 (34.86)		91 (50)	45 (46.39)	46 (54.12)	
	**Overall**	**Survived**	**Died**		**Overall**	**Survived**	**Died**	
No navigation	184 (66.43)	116 (67.84)	68 (64.15)	0.60	91 (50)	63 (52.07)	28 (45.9)	0.53
Navigation	93 (33.57)	55 (32.16)	38 (35.85)		91 (50)	58 (47.93)	33 (54.1)	
	**Overall**	**No Incomplete Ablation**	**Incomplete Ablation**		**Overall**	**No Incomplete Ablation**	**Incomplete Ablation**	
No navigation	184 (66.43)	179 (66.79)	5 (55.56)	0.50	91 (50)	86 (50.29)	5 (45.45)	>0.99
Navigation	93 (33.57)	89 (33.21)	4 (44.44)		91 (50)	85 (49.71)	6 (54.55)	

© 2024 Iwai Y et al. *JAMA Network Open.*

Supplement 5. eFigure 2. Rates of incomplete ablations and recurrences by year, 2011-2022

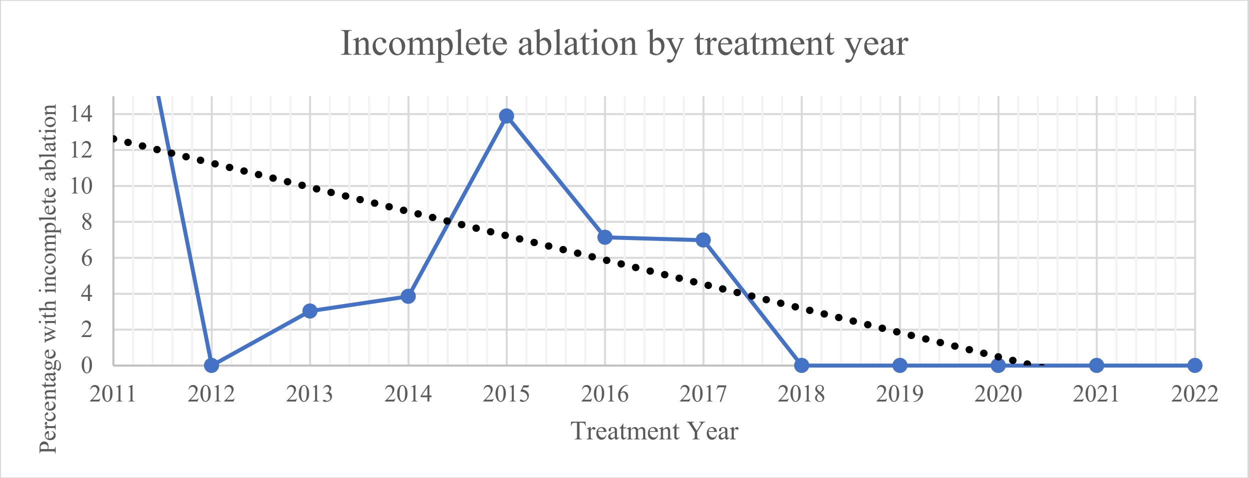

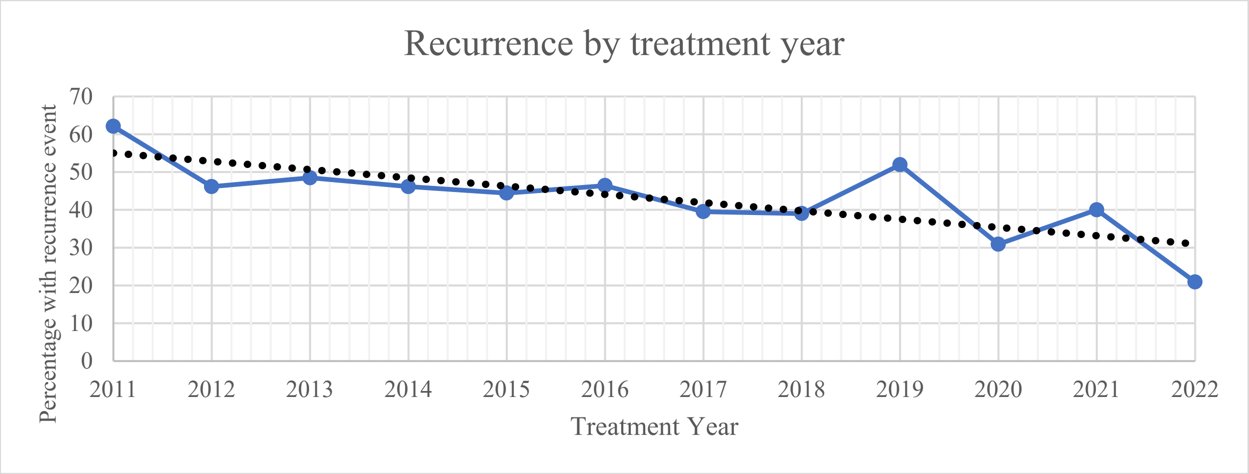

© 2024 Iwai Y et al. *JAMA Network Open.*

Supplement 6. eTable 4. Clinical outcomes of patients undergoing ablative therapy for hepatocellular carcinoma with navigation vs without navigation, 2011-2022

	**All patients (n=467)**	**No Navigation (n=280)**	**Navigation (n=187)**	
**Characteristic**	**No. (%)**	**No. (%)**	**No. (%)**	**p-value**
Orthotopic liver transplant				
No	388 (83.08)	235 (83.93)	153 (81.82)	0.55
Yes	79 (16.92)	45 (16.07)	34 (18.18)	
Readmission				
No	187 (91.22)	81 (87.1)	106 (94.64)	0.08
Yes	18 (8.78)	12 (12.9)	6 (5.36)	
Cause of death				
Cardiac-related	5 (3.01)	4 (4.12)	1 (1.45)	0.26
Liver failure	60 (36.14)	36 (37.11)	24 (34.78)	
OLT-related	3 (1.81)	0 (0)	3 (4.35)	
Other	18 (10.84)	10 (10.31)	8 (11.59)	
Unknown	80 (48.19)	47 (48.45)	33 (47.83)	
Recurrence				
No	272 (58.24)	169 (60.36)	103 (55.08)	0.26
Yes	195 (41.76)	111 (39.64)	84 (44.92)	
Death				
No	298 (63.81)	182 (65)	116 (62.03)	0.56
Yes	169 (36.19)	98 (35)	71 (37.97)	

Abbreviation: OLT = orthotopic liver transplant

© 2024 Iwai Y et al. *JAMA Network Open.*
