# Supplementary material for: Integrating Navigation-Assisted Ablation in the Locoregional Treatment of Hepatocellular Carcinoma
Source: JAMA Netw Open. 2024 Feb 29;7(2):e240694. doi: 10.1001/jamanetworkopen.2024.0694 (PMC10905302; doi:10.1001/jamanetworkopen.2024.0694)
Supplement: Supplement 2. — Data Sharing Statement [file jamanetwopen-e240694-s002.pdf]

Data Sharing Statement

Iwai. Integrating Navigation-Assisted Ablation in the Locoregional Treatment of Hepatocellular Carcinoma. *JAMA Netw Open*. Published online February 29, 2024. doi:10.1001/jamanetworkopen.2024.0694

Data
Data available: No
